# Joint effect of BMI and metabolic status on mortality among adults: a population-based longitudinal study in United States

**DOI:** 10.1038/s41598-024-53229-3

**Published:** 2024-02-02

**Authors:** Feilong Chen, Yunping Shi, Miao Yu, Yuehua Hu, Tao Li, Yijing Cheng, Tao Xu, Junting Liu

**Affiliations:** 1 Department of Epidemiology and Statistics, Institute of Basic Medical Sciences, Chinese Academy of Medical Sciences & School of Basic Medicine, Peking Union Medical College, No.5, Dong dan san tiao, Beijing, 100005 China; 2 Department of Information and Statistics, Beijing Center for Disease Prevention and Control, No. 16 Heping Li Middle Street, Dongcheng District, Beijing, 100013 China; 3 Office of Epidemiology, Chinese Center for Disease Control and Prevention, No. 155 Changbai Road Changping District, Beijing, 102206 China; 4 Child Health Big Data Research Center, Capital Institute of Pediatrics, No. 2 Yabao Road, Beijing, 100020 China

**Keywords:** Cardiology, Health care, Risk factors

## Abstract

We explored the joint effects of different metabolic obesity phenotypes on all-cause and disease-specific mortality risk among the American population. Data were obtained from the National Health and Nutrition Examination Survey (NHANES) 1999–2018. Mortality outcome data were from mortality files linked to National Death Index record and follow-up information was up to December 31, 2019. 50,013 participants were finally included. Four metabolic obesity phenotypes were defined based on obesity and metabolic status: metabolically healthy obese (MHO), metabolically unhealthy obese (MUO), metabolically healthy non-obese (MHNO), and metabolically unhealthy non-obese (MUNO). Population-weighted Cox proportional hazards models were used to explore the all-cause and disease-specific mortality risk of metabolic obesity phenotypes. The all-cause mortality risk of MUO and MUNO was significantly higher than MHNO. MUNO was associated with a significantly increased risk of death from heart disease (HR: 1.40, 95% CI 1.16–1.70), hypertension (HR: 1.68, 95% CI 1.34–2.12), diabetes (HR: 2.29, 95% CI 1.67–3.15), and malignant neoplasms (HR:1.29, 95% CI 1.09–1.53). Metabolic unhealth significantly increased the risk of all-cause mortality, regardless of obesity status. Among individuals with metabolic unhealthy status, obesity significantly reduced the risk of all-cause mortality (HR: 0.91, 95% CI 0.85–0.98). Our study highlights the importance of identifying and characterizing metabolic obesity phenotypes in obese and metabolically abnormal patients, as well as healthy adults. Comprehensive evaluation of obesity and metabolic status is necessary to adopt appropriate interventions and treatment measures and maximize patient benefit.

## Introduction

Obesity has become a critical public health problem worldwide. The World Health Organization (WHO) estimates that the global number of individuals with obesity has exceeded 600 million^1^. The reported age-adjusted prevalence of obesity in the United States (US) was 37.7% (95% confidence interval CI 36.1–39.7%) during 2013–2014 and has been continuously increasing each year^2^. Numerous epidemiological studies have established strong associations between obesity and non-communicable chronic diseases^3^, metabolic abnormalities^4,5^, and increased mortality rates^6,7^. A systematic review of 239 prospective studies demonstrated that overweight and obesity were significantly associated with increased mortality rates^8^. These findings have been corroborated in numerous studies and systematic reviews reporting that obese individuals have a significantly higher risk of cardiovascular disease^9^, type 2 diabetes^10^, malignant tumors^11^, and metabolic abnormalities, such as hypertriglyceridemia^12^ and insulin resistance^13,14^, compared with individuals of normal weight.

However, not all obese people have metabolic problems; on the other hand, a significant proportion of individuals with normal weight, those who were usually considered healthy, might develop metabolic abnormalities as well. Studies have indicated that approximately 30% of obese individuals in Europe have relatively healthy metabolic profiles, with normal glucose tolerance, blood pressure, and lipid levels^15^. The WHO estimates that approximately 200 million people worldwide fall into the category of "obese but metabolically healthy"^16^. Researchers have defined this phenotype as metabolically healthy obesity (MHO). A study using National Health and Nutrition Examination Survey (NHANES) data from 1999 to 2018 found that the standardized prevalence of MHO in the US population increased significantly from 3.2% (95% CI 2.6–3.8%) during 1999–2002 to 6.6% (95% CI 5.3–7.9%) during 2015–2018^17^. Some researchers consider MHO to be a benign condition^15^, but this remains controversial, and a considerable number of studies refute this claim. A large-scale population-based study in Korea found that obese individuals, regardless of metabolic status, had an increased risk of all-cause mortality and cardiovascular events in the long term^18^. Researchers have found that normal weight individuals with significant metabolic abnormalities have lower insulin sensitivity, higher oxidative stress levels, and higher blood pressure levels similar to those observed in obese individuals^19,20^. Epidemiological studies have also suggested that in Asian populations, people who are metabolically unhealthy and non-obese (MUNO) have significantly higher risks of all-cause mortality and non-fatal and fatal cardiovascular events compared with metabolically healthy non-obese (MHNO) individuals^18,21^.

It is important to fully consider both obesity and metabolic status, as well as their combined effects, when estimating mortality risks. Previous studies have predominantly focused on the health effects of the MHO phenotype, with results showing considerable heterogeneity. Furthermore, there is limited research on the mortality risks associated with other metabolic obesity phenotypes in the US population. We aimed to address these knowledge gaps using large-scale survey data from NHANES, which are representative of the US population, to investigate the joint effects of obesity and metabolic status.

## Results

### Baseline characteristics

Among 50,013 participants, there were 24,141 men and 25,872 women. 45,184 participants with complete information on main evaluation indicators and covariables were included in the analysis model. The MUNO phenotype group was the largest (n = 16,966), and the MHO (n = 5063) was the smallest (Table 1). The SBP, DBP, FPG, and TG values of metabolically unhealthy participants were significantly higher than those of metabolically healthy individuals, but HDL-C levels were significantly lower. There were significant differences in the distribution of demographic characteristics (including age, sex, race, family income, and education level) among the different metabolic obesity phenotypes (p < 0.001).

**Table 1:** Baseline characteristics of participants from 10 survey cycles of NHANES 1999–2018.

Variables	MUO	MUNO	MHO	MHNO	*p* value
Total	*n* = 13,471	*n* = 16,966	*n* = 5063	*n* = 14,513	< 0.0001
BMI, kg/m^2^	36.19 ± 5.95	25.68 ± 2.78	35.02 ± 5.39	24.48 ± 2.93	< 0.0001
SBP, mmHg	129.55 ± 19.05	130.06 ± 21.55	115.58 ± 8.75	112.38 ± 9.49	< 0.0001
DBP, mmHg	74.07 ± 14.19	72.52 ± 14.96	69.02 ± 10.50	67.60 ± 10.06	< 0.0001
FPG, mg/dL	117.63 ± 45.51	107.75 ± 39.75	96.27 ± 7.84	93.46 ± 8.39	< 0.0001
HDL-C, mg/dL	44.49 ± 12.94	50.39 ± 16.80	56.33 ± 12.19	61.63 ± 14.66	< 0.0001
TG, mg/dL	172.77 ± 147.20	155.85 ± 127.63	90.72 ± 30.43	80.67 ± 29.68	< 0.0001
Age, year					< 0.0001
20–40	3679 (27.31)	4063 (23.95)	2146 (42.39)	7270 (50.09)	
41–60	4819 (35.77)	5066 (29.86)	1713 (33.83)	4419 (30.45)	
≥ 60	4973 (36.92)	7837 (46.19)	1204 (23.78)	2824 (19.46)	
Gender					< 0.0001
Male	5970 (44.32)	9119 (53.75)	2021 (39.92)	7031 (48.45)	
Female	7501 (55.68)	7847 (46.25)	3042 (60.08)	7482 (51.55)	
Race					< 0.0001
Non-hispanic white	5679 (42.16)	7660 (45.15)	1903 (37.59)	6685 (46.06)	
Non-hispanic black	3289 (24.42)	3000 (17.68)	1551 (30.63)	2646 (18.23)	
Hispanic	3827 (28.41)	4438 (26.16)	1322 (26.11)	3434 (23.66)	
Other	676 (5.02)	1868 (11.01)	287 (5.67)	1748 (12.04)	
Family income to poverty ratio					< 0.0001
≤ 1.30	4038 (29.98)	4786 (28.21)	1368 (27.02)	3467 (23.89)	
> 1.30	8210 (60.95)	10,416 (61.39)	3211 (63.42)	9688 (66.75)	
Unknown	1223 (9.08)	1764 (10.40)	484 (9.56)	1358 (9.36)	
Education					< 0.0001
Less than high school	7326 (54.38)	9402 (55.42)	2340 (46.22)	6017 (41.46)	
High school graduates or GED	4055 (30.10)	4274 (25.19)	1672 (33.02)	4143 (28.55)	
Some college or above	2078 (15.43)	3260 (19.21)	1047 (20.68)	4336 (29.88)	
Unknown	12 (0.09)	30 (0.18)	4 (0.08)	17 (0.12)	
Smoking status					< 0.0001
Non-smoker	7215 (53.56)	8577 (50.55)	3061 (60.46)	8469 (58.35)	
Former smoker	3684 (27.35)	4491 (26.47)	1221 (24.12)	3007 (20.72)	
Current smoker	2572 (19.09)	3898 (22.98)	781 (15.43)	3037 (20.93)	
Drink status					< 0.0001
Non-drinker	6162 (45.74)	7156 (42.18)	2298 (45.39)	5450 (37.55)	
Alcohol drinker	7309 (54.26)	9810 (57.82)	2765 (54.61)	9063 (62.45)	
Congestive heart failure					< 0.0001
Yes	661 (4.91)	584 (3.44)	152 (3.00)	205 (1.41)	
No	12,810 (95.09)	16,382 (96.56)	4911 (97.00)	14,307 (98.59)	
Coronary heart disease					< 0.0001
Yes	716 (5.32)	881 (5.19)	146 (2.88)	316 (2.18)	
No	12,755 (94.68)	16,085 (94.81)	4917 (97.12)	14,196 (7.82)	
Angina/angina pectoris					< 0.0001
Yes	563 (4.18)	538 (3.17)	112 (2.21)	211 (1.45)	
No	12,908 (95.82)	16,428 (96.83)	4951 (97.79)	14,301 (98.55)	
Heart attack					< 0.0001
Yes	777 (5.77)	874 (5.15)	167 (3.30)	329 (2.27)	
No	12,694 (94.23)	16,092 (94.85)	4896 (96.70)	14,183 (97.73)	
Stroke					< 0.0001
Yes	632 (4.69)	780 (4.60)	142 (2.80)	266 (1.83)	
No	12,839 (95.31)	16,186 (95.40)	4921 (97.20)	14,246 (98.17)	
Emphysema					< 0.0001
Yes	274 (2.03)	434 (2.56)	75 (1.48)	190 (1.31)	
No	13,197 (97.97)	16,532 (97.44)	4988 (98.52)	14,322 (98.69)	
Chronic bronchitis					< 0.0001
Yes	1068 (7.93)	913 (5.38)	339 (6.70)	578 (3.98)	
No	12,403 (92.07)	16,053 (94.62)	4724 (93.30)	13,934 (96.02)	
Cancer or malignancy					< 0.0001
Yes	1266 (9.40)	1933 (11.39)	397 (7.84)	1006 (6.93)	
No	12,205 (90.60)	15,033 (88.61)	4666 (92.16)	13,506 (93.07)	

Continuous variables were expressed as weighted mean ± SD, and comparison between metabolic obesity phenotypes were conducted based on weighted linear model; Categorized variables were expressed as number (percentage in colons) and compared using Rao-Scott χ^2^ tests.

*MUO* metabolically unhealthy obesity, *MUNO* metabolically unhealthy non-obesity, *MHO* metabolically healthy obesity, *MHNO* metabolically healthy non-obesity, *BMI* body mass index, *SBP* systolic blood pressure, *DBP* diastolic blood pressure, *FPG* fast plasma glucose, *TG* triglyceride, *HDL*-*C* high-density lipoprotein cholesterol, *GED* general education development.

### Risk of mortality with different metabolic obesity phenotypes

**Figure 1:**
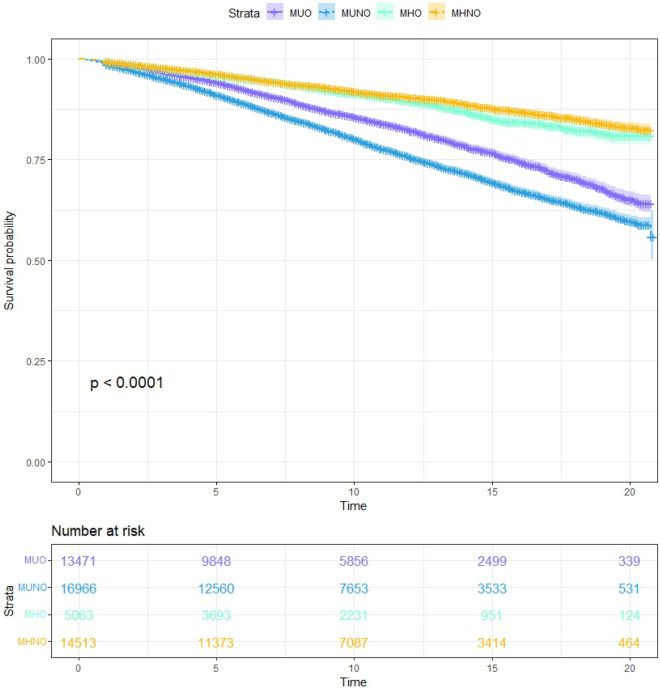
K-M survival curves of different metabolic obesity phenotypes.

According to Table 2, the weighted median and inter-quartile range (IQR) follow-up time for four metabolic obesity phenotypes were 8.75 (9.33) years for MUO, 9.83 (9.67) years for MUNO, 8.50 (9.58) years for MHO, and 9.58 (9.58) years for MHNO, respectively, with a total of 7544 deaths. The distribution of follow-up time for four metabolic obesity phenotypes was shown in Supplementary Fig. 1. There were significant differences in survival status among participants with different metabolic obesity phenotypes (Fig. 1). Among them, the MUNO group had the highest weighted all-cause mortality rate (16.38/person year) and the MHNO group had the lowest rate (5.66/person year). For deaths from heart disease, hypertension, and malignant neoplasm, the weighted mortality rates, which were calculated using constructed 20-year sampling weights to ensure the representativeness of overall American population, in the MUNO phenotype were higher than those in the other groups. For diabetes mortality, the MUO group had the highest risk of mortality.

**Table 2:** Mortality of different metabolic obesity phenotypes.

	MUO	MUNO	MHO	MHNO	Total
Weighted median year of follow-up (IQR)	8.75 (9.33)	9.83 (9.67)	8.50 (9.58)	10.00 (9.67)	9.58 (9.58)
All-cause mortality					
n (%)	2,099 (12.58)	3,735 (16.60)	456 (6.75)	1,254 (5.84)	7544 (11.04)
Mortality rate/1000 person-year	13.35	16.38	7.30	5.66	11.13
Heart disease mortality					
n (%)	593 (3.58)	949 (3.90)	112 (1.62)	286 (1.23)	1940 (2.71)
Mortality rate/1000 person-year	3.80	3.84	1.76	1.20	2.73
Hypertension mortality					
n (%)	371 (1.99)	639 (2.43)	52 (0.68)	160(0.61)	1222 (1.55)
Mortality rate/1000 person-year	2.12	2.40	0.73	0.59	1.56
Diabetes mortality					
n (%)	354 (2.12)	405 (1.49)	47 (0.61)	77 (0.32)	883 (1.18)
Mortality rate/1000 person-year	2.25	1.47	0.65	0.31	1.19
Malignant neoplasms mortality					
n (%)	484 (2.89)	789 (3.77)	121 (1.78)	314 (1.52)	1708 (2.60)
Mortality rate/1000 person-year	3.07	3.72	1.93	1.47	2.62

*IQR* inter-quartile range, *MUO* metabolically unhealthy obesity, *MUNO* metabolically unhealthy non-obesity, *MHO* metabolically healthy obesity, *MHNO* metabolically healthy non-obesity.

Mortality rate and mortality rate/1000 person-year were weighted based on NHANES merged MEC exam weight to ensure the representativeness of American population.

Compared with the MHNO phenotype, the all-cause mortality risks of the MUO (HR: 1.28, 95% CI 1.16–1.41) and MUNO groups (HR: 1.39, 95% CI 1.27–1.52) were significantly increased after adjusting for confounders; there was no significant difference between the MHO and MHNO phenotypes (Table 3). The risks of various causes of mortality in the MUNO group were significantly higher than those in the MHNO group, even after adjusting for the covariates. For participants with the MUO phenotype, the risks of heart disease, hypertension, and diabetes mortality were 1.55 (95% CI 1.27–1.88), 1.67 (95% CI 1.30–2.15), and 3.86 (95% CI 2.70–5.51) times higher than those in the MHNO group, respectively. There was no significant difference in the risk of malignant neoplasm mortality between MUO and MHNO phenotypes.

**Table 3:** All-cause and specific disease death risk of metabolic obesity phenotypes.

	MHNO	MUO	MUNO	MHO
All-cause mortality				
Model 1	1 (ref)	2.40 (2.17, 2.65)*	2.89 (2.66 3.15)*	1.32 (1.15, 1.51)*
Model 2	1 (ref)	1.28 (1.16, 1.41)*	1.39 (1.27, 1.52)*	1.10 (0.96, 1.26)
Heart disease mortality				
Model 1	1 (ref)	3.23 (2.66, 3.93)*	3.21 (2.67, 3.86)*	1.50 (1.11, 2.02)*
Model 2	1 (ref)	1.55 (1.27, 1.88)*	1.40 (1.16, 1.70)*	1.19 (0.88, 1.62)
Hypertension mortality				
Model 1	1 (ref)	3.64 (2.90, 4.56)*	4.05 (3.26, 5.05)*	1.26 (0.84, 1.91)
Model 2	1 (ref)	1.67 (1.30, 2.15)*	1.68 (1.34, 2.12)*	1.00 (0.65, 1.55)
Diabetes mortality				
Model 1	1 (ref)	7.46 (5.37, 10.38)*	4.79 (3.57, 6.41)*	2.18 (1.36, 3.47)*
Model 2	1 (ref)	3.86 (2.70, 5.51)*	2.29 (1.67, 3.15)*	1.94 (1.19, 3.17)*
Malignant neoplasms mortality				
Model 1	1 (ref)	2.10 (1.72, 2.58)*	2.52 (2.15, 2.96)*	1.33 (1.02, 1.73)*
Model 2	1 (ref)	1.21 (0.99, 1.49)	1.29 (1.09, 1.53)*	1.12 (0.85, 1.49)

Model 1 did not adjust any confounders; Model 2 adjusted for age, gender, race, smoking status, drinking habit, socioeconomic status and education level.

*MUO* metabolically unhealthy obesity, *MUNO* metabolically unhealthy non-obesity, *MHO* metabolically healthy obesity, *MHNO* metabolically healthy non-obesity. *Statistically significant.

### Subgroup analysis and sensitivity analysis

**Figure 2:**
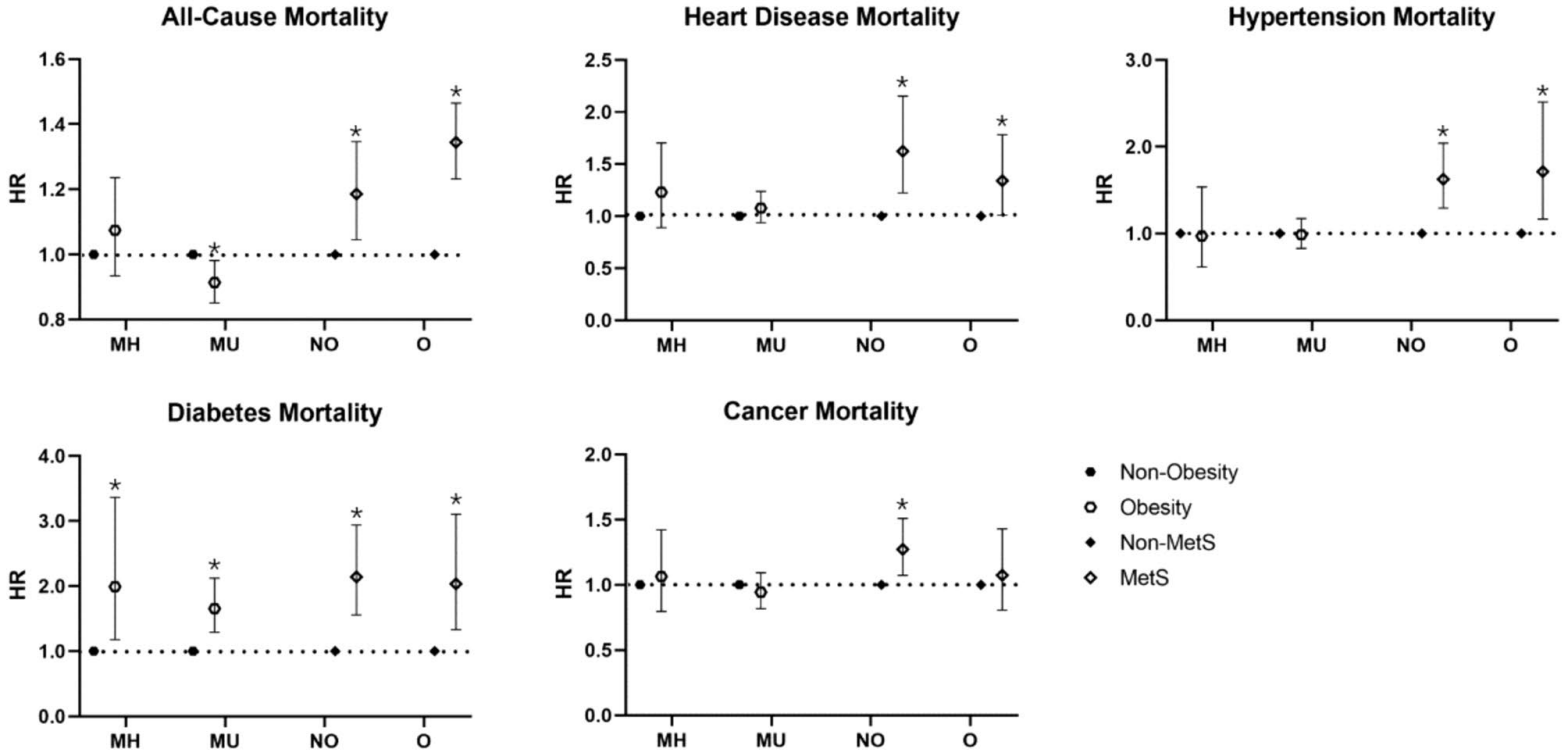
Death risk of metabolic obesity phenotypes stratified by metabolic syndrome and obesity categories. *MH* metabolically healthy, *MU* metabolically unhealthy, *NO* non-obesity, *O* obesity, *MetS* metabolic syndrome. HRs were calculated based on weighted cox regression model after adjusting for age, gender, race, smoking status, drinking habit, socioeconomic status and education level. p for interaction was calculated based on Wald test. All-cause mortality, p for interaction = 0.025; Heart disease mortality, p for interaction = 0.644; Hypertension mortality, p for interaction = 0.984; Diabetes mortality, p for interaction = 0.601; Cancer mortality, p for interaction = 0.257. *Statistically significant.

In this study, interaction and subgroup analysis were carried out according to metabolic status and BMI (Fig. 2). The results suggested that BMI modified the risk of all-cause death of metabolic abnormalities in the fully adjusted model (p for interaction = 0.025). Among participants with a metabolically healthy phenotype, obesity increased the risk of diabetes mortality but had no significant effect on all-cause mortality or other causes of death. For those with metabolic unhealthy status, we found that obesity had a protective effect on the risk of all-cause death (HR: 0.91, 95% CI 0.85–0.98) but significantly increased the risk of diabetes mortality (HR: 2.14, 95% CI 1.56–2.94). However, metabolic abnormalities significantly increased the risk of death from all causes and multiple causes in non-obese people; similar effects of metabolic abnormalities could be seen in obese people (except for the risk of death owing to malignant tumors).

Subgroup analysis was conducted stratified by age, sex, and race (Table 4). Compared with the reference MHNO phenotype, the MUO and MUNO phenotypes had a higher risk of all-cause mortality among adults aged 20–40 years. The MHO phenotype also increased the risk of death in adults aged 40–60 years; only the MUNO phenotype increased the risk of death in people over 60 years old. For participants of different sexes, the effects of metabolic phenotypes on the risk of all-cause mortality were similar to those of the general public. The risk of mortality varied greatly among different race groups. The association between metabolic obesity phenotypes and mortality was not statistically significant for Hispanic people.

**Table 4:** Subgroup analysis of all-cause mortality risk of metabolic obesity phenotypes stratified by age, gender and race.

	MHNO	MUO	MUNO	MHO
Age				
20–40	1 (ref)	2.04 (1.34, 3.10)*	1.63 (1.08, 2.45)*	1.43 (0.83, 2.45)
40–60	1 (ref)	2.00 (1.61, 2.49)*	1.54 (1.24, 1.90)*	1.43 (1.03, 1.99)*
≥ 60	1 (ref)	1.00 (0.90, 1.11)	1.23 (1.12, 1.35)*	0.93 (0.82, 1.06)
Gender				
Male	1 (ref)	1.27 (1.11, 1.46)*	1.26 (1.11, 1.43)*	1.11 (0.92, 1.34)
Female	1 (ref)	1.30 (1.13, 1.50)*	1.57 (1.39, 1.76)*	1.09 (0.88, 1.36)
Race				
Non-hispanic white people	1 (ref)	1.28 (1.14, 1.45)*	1.42 (1.27, 1.57)*	1.08 (0.92, 1.28)
Non-hispanic black people	1 (ref)	1.05 (0.87, 1.27)	1.34 (1.12, 1.60)*	1.05 (0.82, 1.34)
Hispanic people	1 (ref)	1.22 (0.95, 1.57)	1.12 (0.91, 1.37)	1.00 (0.71, 1.41)
Other	1 (ref)	2.35 (1.39, 3.95)*	1.40 (0.89, 2.19)*	1.75 (0.57, 5.41)

HRs were calculated based on weighted cox regression model after adjusting for age, gender, race, smoking status, drinking habit, socioeconomic status and education level.

*MUO* metabolically unhealthy obesity, *MUNO* metabolically unhealthy non-obesity, *MHO* metabolically healthy obesity, *MHNO* metabolically healthy non-obesity. *Statistically significant.

## Discussion

We defined four metabolic obesity phenotypes to distinguish obesity from its usual metabolic consequences and explore their joint effects on mortality. The study findings showed that the MUNO phenotype had the highest risk of all-cause mortality, followed by the MUO phenotype, and both had a significantly increased risk of mortality compared with the MHNO group. In contrast, the MHO phenotype showed no significant difference in risk compared with MHNO. Specifically, we found that obesity reduced the risk of all-cause mortality in people with metabolic abnormalities.

In recent years, several studies have focused on the mortality risk of metabolic obesity phenotypes among different populations. Our study was consistent with several large-scale prospective population studies conducted in multiple countries^22–25^. A community survey in the United Kingdom found that the risk of all-cause and cardiovascular disease mortality was significantly higher in participants with at least two metabolic abnormalities (regardless of obesity status) than in those with MHNO phenotype^23^. Another systematic review based on 13 cohort studies in European and American countries reached a similar conclusion^26^.

There is a view that the MHO phenotype does not exist as a distinct metabolic obesity phenotype but is rather a transient metabolic state that will gradually develop into MUO^27,28^. Researchers have found a significant association between the severity and duration of obesity and the incidence of metabolic abnormalities^29^, and individuals with the MHO phenotype have a higher risk of coronary heart disease, cerebrovascular disease, and heart failure than those with the MHNO phenotype^30^. Another view holds that MHO is a benign state and refers to this metabolic phenotype as "benign obesity"^15^. Population-based studies have reported that the risk of all-cause mortality in participants with the MHO phenotype showed no significant difference from that in participants with the MHNO phenotype, and a protective effect of the MHO phenotype on mortality has even been found in some studies. For example, Ortega et al. found that the risk of all-cause and cardiovascular disease mortality in individuals with MHO was 30–50% lower than that in individuals with the MUO phenotype^15^. In our study, we did not find any difference in the risk of all-cause mortality, heart disease, and hypertension mortality between the MHO phenotype and the control group (MHNO), which was consistent with the findings of Ortega et al.

However, we also find that MHO significantly increased the risk of diabetes mortality, which might be owing to misclassification of the MHO phenotype. One study suggested that 30% of BMI-defined obese participants were classified as MHO based on fasting blood glucose levels, but impaired glucose tolerance or even type 2 diabetes was detected when participants underwent oral glucose tolerance tests^31^, indicating that one-third of obese patients who were considered metabolically healthy actually had glucose metabolism disorders. BMI might lead to misclassification of obesity owing to its lack of sensitivity in distinguishing between fat and muscle tissue^32^, classifying as obese a healthy person with well-developed muscles or a person with relatively low visceral fat accumulation but a BMI above normal. It has been reported that body fat percentage is superior to BMI in identifying participants with impaired glucose tolerance^33^. Thus, indicators to more accurately evaluate obesity are necessary.

Another important finding is that weighted all-cause mortality and mortality owing to heart disease, hypertension, diabetes, and malignant tumors were higher in participants with the MUNO phenotype than in those with the MUO phenotype. Stratified analysis showed that regardless of obesity status, metabolic abnormalities significantly increased the risk of all-cause mortality as well as mortality rates from heart disease, hypertension, and diabetes, after adjusting for confounding factors. Obesity is significantly associated with the reduced risk of all-cause mortality in participants with metabolic abnormalities, which can explain why the risk of all-cause mortality in the MUNO phenotype was higher than that in the MUO phenotype. This result supports the existence of the “obesity paradox,” which has been reported in several studies^34,35^. Among people aged 60 years and older, only the risk of all-cause mortality in the MUNO phenotype was significantly increased; the risk of all-cause mortality in the MUO and MHO groups did not differ significantly from that in the MHNO phenotype. Better nutritional status, healthier cardiopulmonary function, and more medical interventions might be among the reasons explaining the obesity paradox. However, this does not mean that weight gain should be encouraged to reduce the risk of all-cause mortality because metabolic and cardiovascular complications are not the only adverse effects of obesity on health. In fact, the NCEP-ATP III considers overweight and obesity to be direct targets for metabolic syndrome intervention^36^. Additionally, the obesity paradox has also been widely questioned in recent years^37^. One viewpoint is that obesity is not the cause of obese people's reduced risk of mortality. Compared with emaciated people, obesity may not protect humans and reduce the risk of death. On the one hand, most people were greatly ill before they dead, such as cancer, and caused great losses in weight, while people who were still in normal weight or overweight at the time of death might not suffer from disease, resulting in a lower risk of death. On the other hand, patient's metabolic profiles could be different, even for the same BMI, and this profile could influence survival.

Currently, several popular hypotheses have been proposed to explain the differences in mortality risk among individuals with different metabolic phenotypes. First, lifestyle characteristics are important factors. Matheson et al. found that individuals who adopt healthy lifestyle habits (including moderate alcohol consumption, non-smoking, at least 30 min of exercise per day, and daily consumption of fruits and vegetables) had no significant difference in mortality rates compared with individuals who had normal BMI, even if they were obese^38^. It has been shown that lifestyle can change the body's energy metabolism processes, and exercising while consuming a high-energy diet can increase fatty acid oxidation. Additionally, insulin sensitivity is positively correlated with the ability to extract energy from fat tissue^39^. Second, visceral and ectopic fat accumulation and impaired fatty acid supply might also be possible reasons. It is generally believed that body fat distribution and adipose tissue dysfunction in abdominal fat are better predictors of obesity-related metabolic abnormalities than total fat mass itself^40,41^. However, under the current definition of the metabolic obesity phenotype, it is not possible to accurately determine the fat distribution, leading to heterogeneity across studies. Additionally, mechanisms such as insulin resistance, dysregulation of inflammatory regulation, and gut microbiota might also play a role^4,42^.

This study has some limitations. First, it has been shown that the duration of obesity is also an important predictor of metabolic risk in adults^43^, but our study lacked information regarding dynamic changes in BMI. Weight changes could have occurred over the follow-up period. Similar problems also existed in the evaluation of metabolic status. Second, a risk of misclassification was present. On the one hand, BMI lacks sensitivity to distinguish between fat and muscle, which may lead to inaccurate classification of obesity. On the other hand, the definition of metabolic abnormalities in this study relied only on the results of a single blood biochemistry test. Blood composition could be influenced by various short-term exposure factors, which might lead to misclassification of metabolic status. Additionally, we did not focus on specific types of metabolic abnormalities when defining metabolic unhealthy, which may have different effects on long-term risk estimation.

Our study emphasizes the importance of identifying and characterizing metabolic obesity phenotypes and the need to comprehensively evaluate the obesity and metabolic status of individuals, to adopt appropriate early interventions and treatment measures that maximize patient benefit. Our conclusions require further clinical and laboratory research with higher levels of evidence to elucidate the underlying mechanisms.

## Methods

### Participants and data collection

Data were obtained from NHANES, which uses a complex, multi-stage sampling design to ensure representativeness of the US population. Detailed sampling information can be obtained from the NHANES website^44–47^. The present research has been approved by the Ethics Committee^48^.

**Figure 3:**
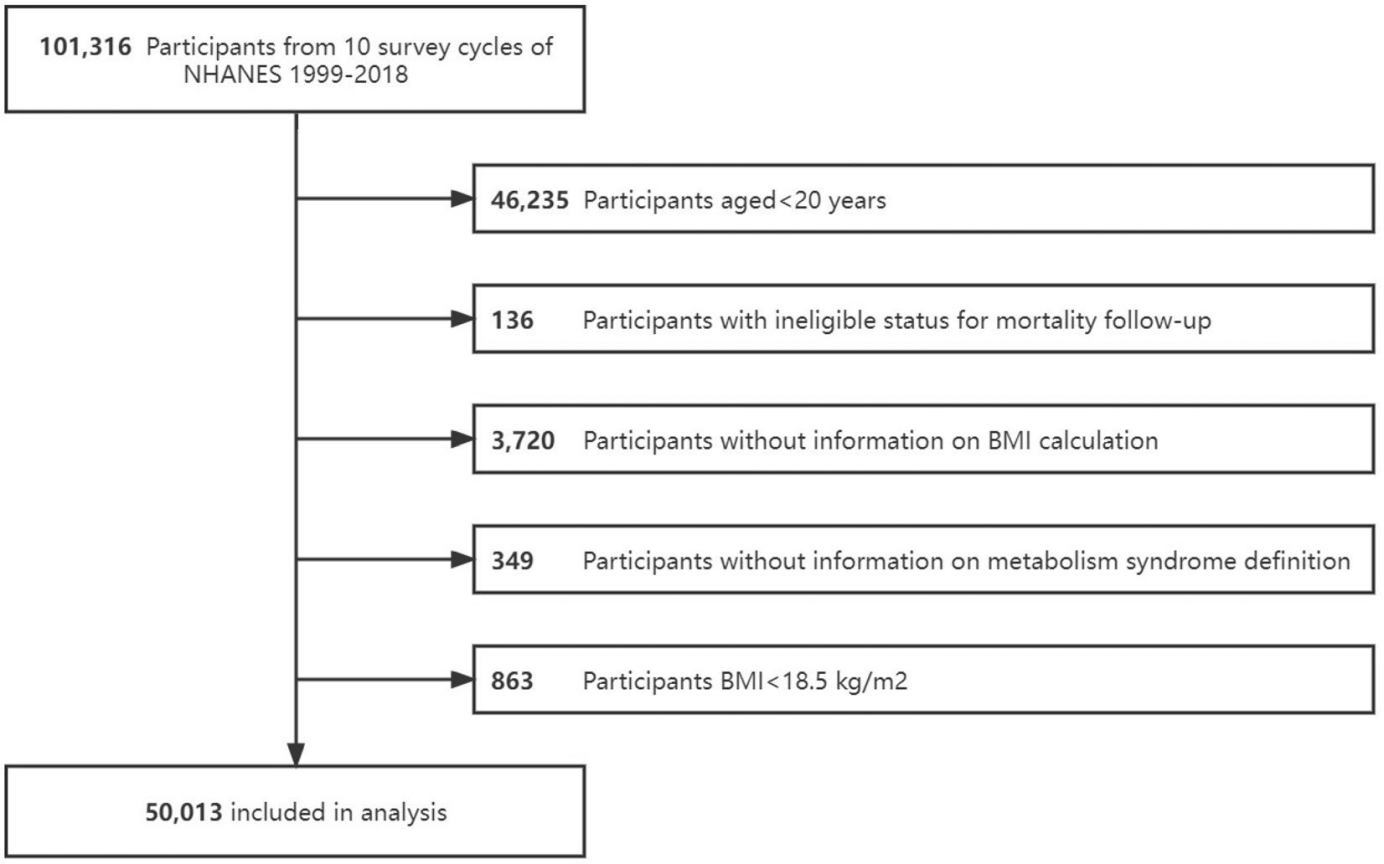
Flow chart of participants selection.

Our study included participants in 10 NHANES cycles from 1999 to 2018, excluding those who were aged less than 20 years (n = 46,325), those who were ineligible for mortality follow-up (n = 136), those who lacked essential information for calculating body mass index (BMI) and defining metabolic status (n = 4069), and those with BMI < 18.5 kg/m^2^ (n = 863). Finally, 50,013 participants were enrolled in this study (Fig. 3). Participants with missing information on covariates (n = 4829) would be removed from the regression analysis. Sensitivity analysis using indicator variables for missing data was also performed to avoid potential selection bias.

All participants signed an informed consent form.

### Data collection

Information on demographic characteristics, lifestyle habits, and results of physical examinations and laboratory tests was collected. Demographic characteristics included age, gender, race, family income status, and education level. Smoking and drinking status, as well as medication history, were also recorded. The data above were self-reported by the participants themselves through face-to-face household interviews. Physical examination and blood sample collection were carried out in standardized mobile medical centers (MECs) by trained nurses^49^. Researchers developed strict quality control measures and standard operating procedures to ensure the reliability of data collection. Height and weight measurements were used to calculate the BMI. Blood pressure (BP) and serological indicators (fasting plasma glucose, triglyceride, and high-density lipoprotein cholesterol) were used to define the metabolic status.

### Metabolic obesity phenotype criteria

BMI ≥ 30 kg/m^2^ was defined as obesity^50^. Metabolic status was defined according to the standardized definition proposed by National Cholesterol Education Program—Adult Treatment Panel III (NCEP-ATP III)^36^. Participants were defined as metabolic unhealthy if they had at least one of the four following components: (1) elevated blood pressure (BP; systolic BP [SBP] ≥ 130 mm Hg, diastolic BP [DBP] ≥ 85 mm Hg); (2) elevated fasting plasma glucose (FPG ≥ 110 mg/dL); (3) reduced high-density lipoprotein cholesterol (HDL-C < 40 mg/dL for men and < 50 mg/dL for women); or (4) elevated triglycerides (TG ≥ 150 mg/dL). Waist circumference was not included in the definition criteria owing to its strong collinear relationship with BMI^17^. Non-obese participants without metabolic abnormalities were defined as MHNO, and those with metabolic unhealthy were defined as MUNO. Participants with obesity who met the metabolic unhealthy criteria were classified as metabolically unhealthy obese (MUO) and those without metabolic problems were classified as MHO.

### Outcomes and follow-up time

The National Center for Health Statistics (NCHS) links data collected from NHANES 1999–2018 with death certificate records from the National Death Index and releases a restricted-use version of the linked mortality files (LMF) to the public, which are available through the NCHS Research Data Center website^51,52^. The public-use LMF provides mortality follow-up data from the date of survey participation through December 31, 2019.

The primary outcome of the study was all-cause mortality. Secondary outcomes included mortality owing to heart disease (International Classification of Diseases Tenth Revision [ICD-10] codes 54–68) and malignant neoplasms (ICD-10 codes 19–43). Additionally, we analyzed the mortality rates of hypertension and diabetes, which are available from the current public-use LMF. Follow-up times were calculated using person-years from the date of the interview to the date of death or the end of the mortality period. Those with no final outcome events as of December 31, 2019 are treated as censored.

### Covariates

We adjusted for demographic characteristics (including age, sex, race, family income, and education level) and lifestyle factors (including smoking and drinking habits) when exploring the association between mortality and different metabolic and obesity phenotype combinations. Race/ethnicity was divided into four categories (Non-Hispanic White, Non-Hispanic Black, Hispanic, and other races). Family income level was calculated by the ratio of household income to poverty ratio (IPR), and IPR greater than 1.3 was defined as a better socioeconomic condition^53^. Education level was categorized into three levels (less than high school, high school graduates or general education development, and some college or above). Smoking status was divided into non-smokers, former smokers, and current smokers. Participants who had drunk alcoholic beverages at least 12 times in the past year were considered to have a history of drinking.

### Statistical analysis

We used SAS 9.4 (SAS Institute, Cary, NC, USA) for data analysis. We constructed 20-year combined MEC complex survey design weights for analysis according to the NHANES analytic guidelines. Continuous variables are described using weighted mean and standard deviation, and comparison between groups are conducted through weighted linear regression analysis. Categorical variables are summarized using number and percentage, and are compared based on Rao-Scott χ^2^ test.

We used Kaplan–Meier survival curves to describe the survival status of different metabolic obesity phenotypes. Cox proportional hazards regression models were applied to estimate the hazard ratios (HRs) and 95% confidence intervals (CIs) of all-cause mortality associated with different metabolic obesity phenotypes. The proportional hazards assumption was examined through goodness-of-fit test and no significant deviation from the assumption was found. Two models were performed. Model 1 was unadjusted; Model 2 was adjusted for age, sex, race, smoking status, drinking habits, socioeconomic status, and education level. Observations with missing information on covariates will be removed from the analytical model. Competing-risks Cox regression analysis^54^ was performed to explore the associations among mortality from heart disease, hypertension, diabetes, malignant neoplasms, and different metabolic obesity phenotypes.

We further investigated the interaction effects of BMI and metabolic status on all-cause and various cause of mortality in the whole-population model. Subsequently, we performed subgroup analyses stratified by metabolic status, obesity groups, and demographic characteristics (age, sex, race), respectively, to explore the relationship among metabolic status, obesity and various mortality outcomes. Interaction and subgroup analysis were both fully adjusted, expect for the stratified factor itself in the subgroup analysis. Several sensitivity analyses were also performed. First, we excluded participants who died with less than 2 years of follow-up to eliminate the effects of death owing to other factors on metabolic obesity phenotypes and mortality associations. Second, participants with existing lung or heart disease and malignant neoplasms at baseline were excluded. Additionally, participants from last two survey cycles (NHANES 2015–2016 and 2017–2018) were excluded due to the short follow-up time. Finally, another newly-proposed metabolic syndrome definitions by the International Diabetes Federation (IDF) Task Force on Epidemiology and Prevention was used to evaluate metabolic status^55^.

Two-sided p ≤ 0.05 was considered statistically significant. All analysis were performed in accordance with the NHANES analytic guidelines^56^.

### Institutional review board statement

The protocols of NHANES were approved by the National Center for Health Statistics and Center for Disease Control and Prevention Ethics Review Board. The reference number can be found on the NHANES website (https://www.cdc.gov/nchs/nhanes/irba98.htm).

### Informed consent

Informed consent was obtained from all participants involved in the study.

### Supplementary Information

Supplementary Tables.

Supplementary Figure 1.

## Data Availability

The datasets used and/or analyzed in the study are available from the NHANES website: (https://www.cdc.gov/nchs/nhanes/index.htm).
