# Supplementary material for: Joint effect of BMI and metabolic status on mortality among adults: a population-based longitudinal study in United States
Source: Sci Rep. 2024 Feb 2;14:2775. doi: 10.1038/s41598-024-53229-3 (PMC10837108; doi:10.1038/s41598-024-53229-3)
Supplement: Supplementary file 1 — Supplementary Tables. [file 41598_2024_53229_MOESM1_ESM.docx]

**Supplementary Table 1.** Baseline 20-year MEC complex sampling weights weighted characteristics of participants from 10 survey cycles of NHANES 1999-2018.

| **Variables** | **MUO** | **MUNO** | **MHO** | **MHNO** | ***p* value ^b^** |
| --- | --- | --- | --- | --- | --- |
| Total | *n*=54,006,840 | *n*=65,078,483 | *n*=21,196,264 | *n*=68,073,452 | <.0001 |
| Mortality |  |  |  |  |  |
| Alive | 47,212,559 | 54,272,702 | 19,764,848 | 64,097,701 |  |
| Dead | 6,794,280 | 10,805,781 | 1,431,416 | 3,975,751 |  |
| Age, year |  |  |  |  | <.0001 |
| 20-40 | 16,202,337 | 18,397,357 | 8,986,150 | 34,108,688 |  |
| 41-60 | 22,636,803 | 23,944,419 | 8,141,043 | 24,161,835 |  |
| ≥60 | 15,167,699 | 22,736,707 | 4,069,072 | 9,802,929 |  |
| Gender |  |  |  |  | <.0001 |
| Male | 25,326,161 | 34,347,074 | 9,116,728 | 31,930,834 |  |
| Female | 28,680,679 | 30,731,410 | 12,079,537 | 36,142,618 |  |
| Race |  |  |  |  | <.0001 |
| Non-Hispanic White | 36,293,943 | 44,660,076 | 13,260,204 | 47,594,094 |  |
| Non-Hispanic Black | 7,152,872 | 6,069,194 | 3,662,105 | 6,400,779 |  |
| Hispanic | 8,053,494 | 8,803,087 | 3,303,993 | 8,802,103 |  |
| Other | 2,506,531 | 5,546,126 | 969,962 | 5,276,476 |  |
| Family income to Poverty ratio |  |  |  |  | <.0001 |
| ≤1.30 | 11,415,846 | 13,142,457 | 4,089,825 | 11,268,027 |  |
| >1.30 | 38,675,295 | 46,246,902 | 15,416,861 | 51,721,875 |  |
| Education |  |  |  |  | <.0001 |
| Less than High School | 25,015,599 | 30,170,255 | 8,162,792 | 22,788,559 |  |
| High School Graduates or GED | 17,947,534 | 18,621,825 | 7,569,982 | 20,071,748 |  |
| Some College or above | 11,005,737 | 16,214,579 | 5,453,647 | 25,144,969 |  |
| Smoking Status |  |  |  |  | <.0001 |
| Non-smoker | 28,359,232 | 32,108,993 | 12,483,899 | 39,397,818 |  |
| Former Smoker | 15,015,656 | 16,725,595 | 5,366,599 | 14,726,840 |  |
| Current Smoker | 10,631,952 | 16,243,895 | 3,345,767 | 13,948,794 |  |
| Drink Status |  |  |  |  | <.0001 |
| Non-Drinker | 22,914,791 | 24,631,913 | 8,985,535 | 22,706,198 |  |
| Alcohol Drinker | 31,092,049 | 40,446,571 | 12,210,730 | 45,367,254 |  |
| Congestive heart failure |  |  |  |  | <.0001 |
| Yes | 2,097,991 | 1,664,228 | 465,367 | 628,241 |  |
| No | 51,908,849 | 63,414,255 | 20,730,897 | 67,443,046 |  |
| Coronary heart disease |  |  |  |  | <.0001 |
| Yes | 2,615,141 | 2,764,770 | 566,578 | 1,257,233 |  |
| No | 51,391,699 | 62,313,713 | 20,629,686 | 66,814,054 |  |
| Angina/angina pectoris |  |  |  |  | <.0001 |
| Yes | 2,082,959 | 1,768,810 | 442,708 | 793,500 |  |
| No | 51,923,881 | 63,309,673 | 20,753,556 | 67,277,787 |  |
| Heart attack |  |  |  |  | <.0001 |
| Yes | 2,625,292 | 2,618,106 | 609,415 | 1,192,715 |  |
| No | 51,381,548 | 62,460,377 | 20,586,849 | 66,878,572 |  |
| Stroke |  |  |  |  | <.0001 |
| Yes | 2,025,773 | 2,253,932 | 453,490 | 860,759 |  |
| No | 51,981,066 | 62,824,551 | 20,742,774 | 67,210,528 |  |
| Emphysema |  |  |  |  | <.0001 |
| Yes | 1,075,137 | 1,497,134 | 262,937 | 719,819 |  |
| No | 52,931,703 | 63,581,349 | 20,933,327 | 67,351,468 |  |
| Chronic bronchitis |  |  |  |  | <.0001 |
| Yes | 4,420,246 | 3,796,043 | 1,542,813 | 2,804,557 |  |
| No | 49,586,594 | 61,282,440 | 19,653,451 | 65,266,730 |  |
| Cancer or malignancy |  |  |  |  | <.0001 |
| Yes | 5467,276 | 7,656,085 | 1,705,912 | 4,921,684 |  |
| No | 48,539,564 | 57,422,399 | 19,490,352 | 63,149,603 |  |
| Abbreviations: MUO, metabolically unhealthy obesity; MUNO, metabolically unhealthy non-obesity; MHO, metabolically healthy obesity; MHNO, metabolically healthy non-obesity; BMI, body mass index; SBP, systolic blood pressure; DBP, diastolic blood pressure; FPG, fast plasma glucose; TG, triglyceride; HDL-C, high-density lipoprotein cholesterol; GED, general education development.  Continuous variables were expressed as weighted mean± SD, and comparison between metabolic obesity phenotypes were conducted based on weighted linear model; Categorized variables were expressed as number (percentage in colons) and compared using Rao-Scott χ^2^ tests. | | | | | |

**Supplemtary Table 2.** Risk of all-cause death with different metabolic obesity phenotypes classified by International Diabetes Federation (IDF) standard.

| Variables | *β* | *t* | *p* Value | HR | Lower 95%CI | Upper 95%CI |
| --- | --- | --- | --- | --- | --- | --- |
| Metabolic phenotype (MHO) | 0.09 | 0.79 | 0.430 | 1.10 | 0.87 | 1.37 |
| Metabolic phenotype (MUNO) | 0.48 | 9.14 | <.001 | 1.62 | 1.46 | 1.79 |
| Metabolic phenotype (MUO) | 0.41 | 6.55 | <.001 | 1.50 | 1.33 | 1.69 |
| Metabolic phenotype (MHNO) | 0 (ref) | / | / | 1 (ref) | / | / |
| Abbreviations: MUO, metabolically unhealthy obesity; MUNO, metabolically unhealthy non-obesity; MHO, metabolically healthy obesity; MHNO, metabolically healthy non-obesity; GED, general education development; HR, Hazard ratio; CI, Confidence interval.  HRs were calculated based on weighted cox regression model after adjusting for age, gender, race, smoking status, drinking status, socioeconomic status and educational level. | | | | | | |

**Supplemtary Table 3.** Risk of all-cause death with different metabolic obesity phenotypes excluding paticipants with fewer than 2 years of follow-up before the main outcome occoured.

| Variables | *β* | *t* | *p* Value | HR | Lower 95%CI | Upper 95%CI |
| --- | --- | --- | --- | --- | --- | --- |
| Metabolic phenotype (MHO) | 0.08 | 1.00 | 0.321 | 1.08 | 0.93 | 1.25 |
| Metabolic phenotype (MUNO) | 0.36 | 7.09 | <.001 | 1.43 | 1.29 | 1.57 |
| Metabolic phenotype (MUO) | 0.28 | 5.17 | <.001 | 1.32 | 1.19 | 1.47 |
| Metabolic phenotype (MHNO) | 0 (ref) | / | / | 1 (ref) | / | / |
| Abbreviations: MUO, metabolically unhealthy obesity; MUNO, metabolically unhealthy non-obesity; MHO, metabolically healthy obesity; MHNO, metabolically healthy non-obesity; GED, general education development; HR, Hazard ratio; CI, Confidence interval.  HRs were calculated based on weighted cox regression model after adjusting for age, gender, race, smoking status, drinking status, socioeconomic status and educational level. | | | | | | |

**Supplemtary Table 4.** Risk of all-cause death with different metabolic obesity phenotypes excluding paticipants with existing lung, heart disease and malignant neoplasms.

| Variables | *β* | *t* | *p* Value | HR | Lower 95%CI | Upper 95%CI |
| --- | --- | --- | --- | --- | --- | --- |
| Metabolic phenotype (MHO) | 0.12 | 1.30 | 0.197 | 1.12 | 0.94 | 1.34 |
| Metabolic phenotype (MUNO) | 0.42 | 7.22 | <.001 | 1.52 | 1.35 | 1.70 |
| Metabolic phenotype (MUO) | 0.33 | 5.20 | <.001 | 1.39 | 1.22 | 1.57 |
| Metabolic phenotype (MHNO) | 0 (ref) | / | / | 1 (ref) | / | / |
| Abbreviations: MUO, metabolically unhealthy obesity; MUNO, metabolically unhealthy non-obesity; MHO, metabolically healthy obesity; MHNO, metabolically healthy non-obesity; GED, general education development; HR, Hazard ratio; CI, Confidence interval.  HRs were calculated based on weighted cox regression model after adjusting for age, gender, race, smoking status, drinking status, socioeconomic status and educational level. | | | | | | |

**Supplementary Table 5.** Risk of all-cause death with different metabolic obesity phenotypes excluding participants from NHANES 2015-2016 and 2017-2018 survey cycles.

| Variables | *β* | *t* | *p* Value | HR | Lower 95%CI | Upper 95%CI |
| --- | --- | --- | --- | --- | --- | --- |
| Metabolic phenotype (MHO) | 0.08 | 1.11 | 0.267 | 1.08 | 0.94 | 1.25 |
| Metabolic phenotype (MUNO) | 0.32 | 6.99 | <.001 | 1.38 | 1.26 | 1.51 |
| Metabolic phenotype (MUO) | 0.23 | 4.43 | <.001 | 1.26 | 1.14 | 1.39 |
| Metabolic phenotype (MHNO) | 0 (ref) | / | / | 1 (ref) | / | / |
| Abbreviations: MUO, metabolically unhealthy obesity; MUNO, metabolically unhealthy non-obesity; MHO, metabolically healthy obesity; MHNO, metabolically healthy non-obesity; GED, general education development; HR, Hazard ratio; CI, Confidence interval.  HRs were calculated based on weighted cox regression model after adjusting for age, gender, race, smoking status, drinking status, socioeconomic status and educational level. | | | | | | |

**Supplemtary Table 6.** Risk of all-cause death with different metabolic obesity phenotypes utilizing indicator variables to to deal with missing data.

| Variables | *β* | *t* | *p* Value | HR | Lower 95%CI | Upper 95%CI |
| --- | --- | --- | --- | --- | --- | --- |
| Metabolic phenotype (MHO) | 0.08 | 1.20 | 0.232 | 1.08 | 0.95 | 1.23 |
| Metabolic phenotype (MUNO) | 0.33 | 8.17 | <.001 | 1.40 | 1.29 | 1.51 |
| Metabolic phenotype (MUO) | 0.26 | 5.55 | <.001 | 1.29 | 1.18 | 1.42 |
| Metabolic phenotype (MHNO) | 0 (ref) | / | / | 1 (ref) | / | / |
| Abbreviations: MUO, metabolically unhealthy obesity; MUNO, metabolically unhealthy non-obesity; MHO, metabolically healthy obesity; MHNO, metabolically healthy non-obesity; GED, general education development; HR, Hazard ratio; CI, Confidence interval.  HRs were calculated based on weighted cox regression model after adjusting for age, gender, race, smoking status, drinking status, socioeconomic status and educational level. | | | | | | |
