# Supplementary material for: Joint effect of BMI and metabolic status on mortality among adults: a population-based longitudinal study in United States
Source: Sci Rep. 2024 Feb 2;14:2775. doi: 10.1038/s41598-024-53229-3 (PMC10837108; doi:10.1038/s41598-024-53229-3)
Supplement: Supplementary file 2 — Supplementary Figure 1. [file 41598_2024_53229_MOESM2_ESM.docx]

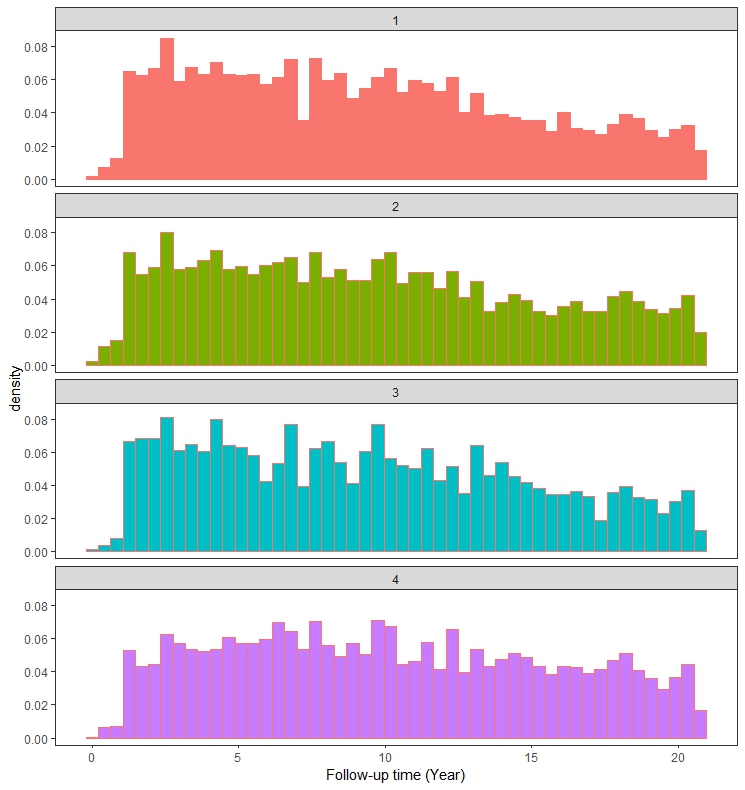


**Supplementary Figure 1.** Distribution of follow-up time for four metabolic obesity phenotypes. 1: Metabolically unhealthy obese; 2: Metabolically unhealthy non-obesity; 3: Metabolically healthy obesity; 4: Metabolically healthy non-obesity.
